# Repurposing Heart Failure Therapies in Pulmonary Arterial Hypertension: Mechanistic Rationale, Translational Evidence, and Clinical Perspectives for SGLT2 Inhibitors and Mineralocorticoid Receptor Antagonists

**DOI:** 10.3390/medicina62071345

**Published:** 2026-07-12

**Authors:** Spyridon Karkoulias, Ioannis Leontsinis, Panagiotis Iliakis, Liza Kallenou, Alexandros Tsiavos, Stergios Soulaidopoulos, Eirini Dri, Eleni Manta, Panayotis K. Vlachakis, Panagiotis Tsioufis, Nikolaos Ktenopoulos, Paschalis Karakasis, Kyriakos Dimitriadis, Polykarpos Christos Patsalis, Christina Chrysohoou, Konstantinos Tsioufis

**Affiliations:** 1 First Cardiology Department, General Hospital of Nikea, Agios Panteleimon Piraeus, 3 D. Mantouvalou Street, 18454 Piraeus, Greece; spyroskarkoulias@gmail.com (S.K.); alextsiavos13@gmail.com (A.T.); 2 First Department of Cardiology, National and Kapodistrian University of Athens, Hippokration General Hospital of Athens, 11527 Athens, Greece; giannisleontsinis@gmail.com (I.L.); soulaidopoulos@gmail.com (S.S.); iredris@hotmail.com (E.D.); vlachakispanag@gmail.com (P.K.V.); ptsioufis@gmail.com (P.T.); nikosktenop@gmail.com (N.K.); dimitriadiskyr@yahoo.gr (K.D.); chrysohoou@usa.net (C.C.); ktsioufis@gmail.com (K.T.); 3 Division of Cardiology, Angiology and Internal Emergency Medicine, Department of Medicine, Knappschaft Kliniken University Hospital Bochum, Ruhr University Bochum, 44892 Bochum, Germany; polykarpos.patsalis@ruhr-uni-bochum.de; 4 Department of Cardiology, KAT General Hospital, 14561 Athens, Greece; lizakallenou025@gmail.com; 5 Second Department of Cardiology, Hippokration General Hospital, 54642 Thessaloniki, Greece; pakar15@hotmail.com

**Keywords:** pulmonary arterial hypertension, sodium–glucose cotransporter 2 inhibitors, mineralocorticoid receptor antagonists, right ventricular dysfunction, pulmonary vascular remodelling

## Abstract

Pulmonary arterial hypertension (PAH) is a progressive and life-threatening condition characterized by elevated pulmonary vascular resistance eventually causing right ventricular failure and premature death. Despite advances in targeted therapies, morbidity and mortality levels remain high, highlighting the need for additional treatment strategies that address the disease’s multifactorial pathophysiology. Attention has recently centred on two pharmacological groups with proven roles in other cardiovascular settings: sodium–glucose cotransporter 2 inhibitors (SGLT2i) and mineralocorticoid receptor antagonists (MRAs). SGLT2 inhibitors have demonstrated robust clinical benefits in heart failure (HF), type 2 diabetes mellitus (T2DM), and chronic kidney disease (CKD), extending survival and reducing hospitalizations. Although their primary actions involve renal glucose and sodium handling, accumulating evidence suggests they may also exert favourable effects on the pulmonary vasculature, endothelial function, and right ventricular performance. In parallel, elevated aldosterone levels have been implicated in vascular remodelling, inflammation, and fibrosis in PAH, suggesting a potential therapeutic role for MRAs. However, the strength and clinical significance of these associations remain under investigation. The aim of this review is to synthesize and critically appraise the current evidence regarding the potential role of SGLT2 inhibitors and MRAs in the treatment of pulmonary arterial hypertension, exploring their mechanistic rationale, preclinical findings, and available clinical data to determine whether these agents may offer additional therapeutic benefit in PAH management.

## 1. Introduction

Pulmonary arterial hypertension (PAH) is characterized by progressive pathological remodelling of the small-calibre pulmonary arteries, leading to increased pulmonary vascular resistance and pulmonary arterial pressure despite normal left atrial pressure [1]. In PAH, chronically increased right ventricular afterload leads to progressive right ventricular dysfunction, right ventricular failure, and ultimately premature death [1,2]. PAH phenotypes include idiopathic, heritable and drug- or toxin-associated PAH, PAH associated with connective tissue disease, HIV infection, portal hypertension, corrected congenital heart disease, schistosomiasis, persistent PH of the newborn and cases in which lesions affect the pulmonary venules/capillaries [3]. Despite significant advances in understanding its underlying pathophysiological mechanisms, PAH remains associated with high mortality, with more than 50% of patients dying within five years of diagnosis [4,5,6]. Mineralocorticoid receptor antagonists are typically used for their potassium-sparing properties; however, despite the well-documented antifibrotic and anti-remodelling effects demonstrated in heart failure populations, current PAH management guidelines do not endorse their use based on these cardioprotective mechanisms [4,6]. Sodium–glucose cotransporter 2 inhibitors (SGLT2i) represent a pharmacological class with well-established cardio-reno-metabolic benefits and are indicated for patients with any phenotype of heart failure, chronic kidney disease, or type 2 diabetes mellitus. Although right ventricular dysfunction is a central feature of PAH, this drug class has not yet been incorporated into current treatment recommendations.

In this review, we critically evaluate the rationale for repurposing sodium–glucose cotransporter-2 inhibitors and mineralocorticoid receptor antagonists as adjunctive therapies in pulmonary arterial hypertension. Rather than merely summarizing available studies, we distinguish direct evidence obtained in Group 1 PAH from findings extrapolated from other forms of pulmonary hypertension, heart failure, diabetes, and chronic kidney disease. We examine the molecular and cellular pathways through which these agents may influence pulmonary vascular remodelling, endothelial dysfunction, inflammation, metabolic dysregulation, and right ventricular adaptation while also appraising the methodological limitations and translational relevance of the current evidence. Finally, we compare the therapeutic profiles of SGLT2 inhibitors and MRAs, identify potential responder phenotypes and safety considerations, and define the principal evidence gaps that should guide future clinical trials.

## 2. Methods

This narrative review was developed through a structured search of PubMed/MEDLINE, Scopus, and Web of Science for studies published up to and including 30 June 2026. The final literature search was completed after the end of June 2026 during preparation of the revised manuscript. Search terms included combinations of “pulmonary arterial hypertension,” “pulmonary hypertension,” “right ventricular failure,” “sodium–glucose cotransporter-2 inhibitors,” “SGLT2 inhibitors,” “empagliflozin,” “dapagliflozin,” “mineralocorticoid receptor antagonists,” “spironolactone,” “eplerenone,” “finerenone,” “aldosterone,” “pulmonary vascular remodelling,” “endothelial dysfunction,” “inflammation,” and “oxidative stress.” Reference lists of relevant articles were also screened to identify additional studies. Eligible publications included experimental studies, translational investigations, observational studies, randomized and non-randomized clinical trials, systematic reviews, major contemporary reviews, clinical guidelines, and registered ongoing trials relevant to SGLT2 inhibition or mineralocorticoid receptor blockade in pulmonary vascular disease. Registered trials without peer-reviewed published results were used only to describe ongoing research activity and were not interpreted as evidence of therapeutic efficacy. Particular emphasis was placed on studies published during the preceding five years, while earlier landmark mechanistic and clinical studies were retained when necessary. Because direct clinical evidence in Group 1 PAH remains limited, studies conducted in other forms of pulmonary hypertension, heart failure, diabetes, and chronic kidney disease were considered only when they provided mechanistically or clinically relevant information. Throughout the review, direct evidence in PAH was distinguished from indirect or extrapolated evidence. The literature was synthesized narratively, with emphasis on biological plausibility, study design, population characteristics, clinical relevance, and major methodological limitations. This review was not conducted as a formal systematic review or meta-analysis.

## 3. Current Treatment Strategies in PAH

Current therapeutic strategies for PAH consist of disease-specific agents alongside general supportive measures [1]. According to current guidelines, treatment decisions in PAH are guided by structured risk assessment. Patients are categorized as low-, intermediate-, or high-risk for 1-year mortality based on clinical, biochemical, functional, and hemodynamic parameters. The primary therapeutic goal is to achieve and maintain the lowest possible risk status, which has been associated with improved long-term outcomes. This risk-stratified approach informs treatment intensity, including the use of monotherapy in selected cases or, more commonly, early combination therapy according to disease severity [4].

### 3.1. PAH-Specific Therapies

Calcium channel blockers remain indicated for patients with idiopathic, heritable, or drug- or toxin-associated PAH who demonstrate a favourable response to acute vasoreactivity testing. Phosphodiesterase type 5 inhibitors promote pulmonary vasodilation and are commonly recommended as part of initial combination therapy with an endothelin receptor antagonist in patients who are not at high risk. In selected patients with substantial comorbidities, initial monotherapy may be considered. Riociguat, a soluble guanylate cyclase stimulator, represents an alternative therapeutic option in eligible patients and should not be combined with phosphodiesterase type 5 inhibitors. Endothelin receptor antagonists exert vasodilatory and antiproliferative effects and constitute a central component of PAH combination therapy. Prostacyclin-pathway therapies have vasodilatory, antiproliferative, antithrombotic, and anti-inflammatory properties, with parenteral prostacyclin therapy playing an essential role in patients at high risk. Selexipag, an oral prostacyclin-receptor agonist, may be added to existing therapy to reduce the risk of clinical worsening [1,2,7]. Sotatercept, a first-in-class activin-signalling inhibitor, has emerged as an important therapeutic advance in PAH by targeting pathways involved in pulmonary vascular remodelling rather than acting solely through vasodilation [2]. In the PULSAR trial, sotatercept reduced pulmonary vascular resistance and improved exercise capacity and biomarkers in patients receiving stable background PAH therapy [8]. More recently, phase III data have shown clinically meaningful benefit across a broader PAH spectrum, including patients with WHO functional class II–III disease and patients with advanced disease receiving maximally tolerated background therapy [8,9]. These benefits have included improvements in exercise capacity and delays in clinical worsening, while data in newly diagnosed PAH further suggest a potential role earlier in the treatment pathway. Importantly, the mechanism of sotatercept has raised the possibility that it may exert disease-modifying effects by addressing pulmonary vascular remodelling [10]. However, confirmation of true disease modification will require longer-term follow-up and further mechanistic studies.

### 3.2. Supportive and Non-PAH-Specific Treatment

For patients who remain at high risk despite maximal medical therapy, lung transplantation should be considered [11,12,13,14]. In addition, advanced support, including inotropic agents and mechanical circulatory assistance, may be necessary for refractory cases or as bridge to lung transplantation [13,15,16].

Supervised exercise training, psychological support, vaccination against SARS-CoV-2, influenza, pneumococcal infection, and respiratory syncytial virus, oxygen supplementation in patients with respiratory failure (PaO_2_ < 60 mmHg), and iron replacement in patients with iron deficiency constitute general supportive measures in patients with pulmonary arterial hypertension (PAH), alongside counselling against pregnancy [1,17]. Fluid retention secondary to right ventricular dysfunction is common in PAH, and diuretic therapy is recommended to manage volume overload. Loop, thiazide, and potassium-sparing diuretics are commonly used in clinical practice for this purpose [1,17].

## 4. SGLT2 Inhibitors in PAH

Sodium–glucose cotransporter-2 inhibitors (SGLT2i) have attracted increasing interest as potential adjunctive therapies in pulmonary arterial hypertension because their cardiovascular actions extend beyond glycosuria and osmotic diuresis. Proposed mechanisms relevant to PAH include improvement in endothelial function and nitric oxide bioavailability, attenuation of oxidative and inflammatory signalling, modulation of mitochondrial and myocardial energetics, and inhibition of pulmonary vascular and right ventricular remodelling [18,19]. However, the supporting evidence is heterogeneous and occasionally conflicting. Most data derive from experimental pulmonary hypertension models, whereas human evidence has largely been extrapolated from patients with diabetes, heart failure, or post-capillary pulmonary hypertension. More recently, observational data in patients labelled as having PAH have emerged, but prospective randomized evidence in hemodynamically confirmed Group 1 PAH remains lacking. Consequently, mechanistic plausibility, experimental efficacy, indirect human haemodynamic effects, and direct clinical evidence should be considered separately [19].

### 4.1. Endothelial and Vasodilatory Effects

SGLT2 inhibitors may promote pulmonary vasodilation through multiple endothelial and vascular mechanisms. These agents may increase nitric oxide bioavailability through a reduction in oxidative stress and improvement in endothelial function. In addition, SGLT2is appear to reduce endothelin-1 expression, thereby potentially attenuating vasoconstriction and pulmonary vascular resistance [20]. Experimental evidence summarized by Luo et al. suggests that diabetic mouse models demonstrated improved pulmonary artery relaxation and reduced vascular remodelling following empagliflozin administration, possibly mediated through enhanced endothelial nitric oxide synthase activity [21]. Collectively, these effects may contribute to reductions in pulmonary arterial pressure and right ventricular afterload.

### 4.2. Anti-Inflammatory and Antioxidative Mechanisms

SGLT2 inhibitors may also attenuate inflammation and cardiac remodelling, both of which contribute to the pathophysiology of PAH. Animal-model data indicate that SGLT2 inhibitors may attenuate pulmonary vascular remodelling in PAH, potentially through anti-inflammatory mechanisms that reduce cytokine release and vascular inflammation—processes that may in turn mitigate endothelial dysfunction and smooth muscle proliferation [22]. SGLT2 inhibitors may also reduce oxidative stress, which has been linked to endothelial damage, dysfunction and remodelling. Moreover, they ameliorate metabolic dysfunction, which is increasingly recognized as a contributing factor to PAH [20,23]. Studies in HIV-associated pulmonary hypertension have further highlighted the importance of inflammatory and endothelial pathways in pulmonary vascular disease. Increased expression of endothelin-1, TNF-α, VEGF, and other pro-inflammatory mediators contributes to vasoconstriction, endothelial dysfunction, and vascular remodelling [24]. ET-1 has vasoconstrictive, proinflammatory, and pro-mitotic characteristics, promoting free radical generation and platelet activation [25]. Inflammatory signalling pathways, notably those involving MMP-9, ICAM-1, and cyclooxygenase-2, have important regulatory roles in pulmonary vascular disease. Evidence from experimental and cardiovascular studies suggests that SGLT2 inhibitors may reduce the expression of ICAM-1, VCAM-1, and several pro-inflammatory interleukins, thereby improving endothelial function [26]. These findings underscore a broader therapeutic principle: targeting cellular and metabolic dysfunction can reverse pulmonary vascular remodelling and right heart failure. This mechanistic rationale aligns with data suggesting that SGLT2 inhibitors, through modulation of myocardial energetics and oxidative stress, may provide benefit in Group 1 PAH, a condition similarly characterized by progressive pulmonary vascular remodelling and right ventricular strain [27]. More direct evidence comes from a monocrotaline-induced PAH model in which dapagliflozin reduced right ventricular systolic pressure and pulmonary vascular remodelling while suppressing activation of the NLRP3 inflammasome and reducing circulating interleukin-1β and interleukin-18 levels. However, combining dapagliflozin with sildenafil did not provide greater haemodynamic or structural benefit than sildenafil alone, suggesting that the mechanistic activity of dapagliflozin may not necessarily translate into additive efficacy when administered alongside established PAH-targeted therapy [28].

### 4.3. Metabolic and Mitochondrial Effects

Beyond their vascular and anti-inflammatory effects, SGLT2 inhibitors may influence mitochondrial energetics, redox balance, and cellular survival [29]. Experimental cardiovascular studies suggest that these agents can improve mitochondrial efficiency, reduce excessive reactive oxygen species generation, preserve adenosine triphosphate production, and limit apoptosis. These mechanisms may be relevant to PAH because both pulmonary vascular cells and the pressure-overloaded right ventricle exhibit metabolic reprogramming, impaired mitochondrial function, and reduced energetic reserve. Improved mitochondrial homeostasis could therefore support endothelial-cell survival and right ventricular adaptation. However, most of this evidence derives from general cardiovascular or metabolic models rather than human PAH tissue, and it remains uncertain whether these cellular effects translate into meaningful improvements in pulmonary hemodynamics or clinical outcomes.

### 4.4. Experimental, Translational, and Clinical Evidence

Direct experimental evidence has produced encouraging but inconsistent findings. In a monocrotaline-induced pulmonary hypertension model, empagliflozin reduced mortality, right ventricular systolic pressure, right ventricular hypertrophy and fibrosis, and pulmonary arterial muscularization, suggesting combined pulmonary vascular and right ventricular effects [30]. Similarly, dapagliflozin attenuated pulmonary vascular remodelling and inflammatory signalling in another monocrotaline model, as discussed above [28]. More recent experimental work suggested that empagliflozin may inhibit pulmonary arterial smooth-muscle-cell proliferation and vascular remodelling through suppression of platelet-derived growth factor receptor-β phosphorylation [31]. However, these findings have not been reproduced consistently. Li et al. found that dapagliflozin failed to improve pulmonary hemodynamics, vascular remodelling, or right ventricular structure and function in either monocrotaline-induced PAH or pulmonary trunk-banding models [32]. Differences in the SGLT2 inhibitor used, dose, treatment timing, experimental model, and severity of established disease may partly explain these divergent results. Overall, the preclinical literature supports biological activity but does not establish a uniform class effect or reliably demonstrate direct right ventricular protection.

Clinical evidence remains limited and is derived mainly from populations without established Group 1 PAH. In a randomized controlled trial of patients with type 2 diabetes and increased cardiovascular risk, six months of dapagliflozin reduced exercise-induced right ventricular systolic pressure and left ventricular filling pressure, while stroke volume index and cardiac index remained unchanged [33]. Although these findings suggest a favourable effect on exercise hemodynamics, the study population did not have hemodynamically confirmed pre-capillary PAH, and pulmonary vascular resistance was not evaluated. The results should therefore be regarded as supportive of biological plausibility rather than as evidence of therapeutic efficacy in PAH. Similar indirect evidence comes from the EMBRACE-HF trial, in which empagliflozin reduced pulmonary artery pressures in patients with heart failure monitored using implanted pulmonary artery pressure sensors [34]. However, this effect was observed in the setting of left-heart disease and may have been related mainly to changes in cardiac filling pressures and volume status. Its relevance to the fixed pulmonary vascular remodelling characteristic of Group 1 PAH consequently remains uncertain.

Despite accumulating indirect evidence that SGLT2 inhibitors may reduce pulmonary pressures in left-heart disease and exercise-related settings, no adequately powered randomized trial has yet demonstrated clinical efficacy in hemodynamically confirmed pre-capillary PAH [35]. Several studies have been registered to evaluate SGLT2 inhibitors directly in PAH, including the DAPAH trial of dapagliflozin (NCT05179356), a completed phase IIa feasibility study of empagliflozin (NCT05493371), and an ongoing study evaluating the effects of empagliflozin on right ventricular function and other clinically relevant outcomes (NCT06992440). Preliminary findings from the completed empagliflozin study have been reported in preprint form; however, because these data have not yet undergone full peer review, they should be interpreted cautiously and should be considered hypothesis-generating rather than evidence of established efficacy. These studies may help clarify the safety, feasibility, haemodynamic effects, and potential clinical relevance of SGLT2 inhibition in PAH. Collectively, these findings provide a rationale for further investigation of SGLT2 inhibitors in PAH. A summary of the principal studies evaluating SGLT2i in pulmonary hypertension is presented in Table 1.

## 5. Mineralocorticoid Receptor Antagonists in PAH

The mineralocorticoid receptor (MR) is a central and highly adaptable transcription factor that influences multiple signalling pathways involved in the pathogenesis of pulmonary hypertension [36]. In pulmonary arterial endothelial cells (PAECs), MR activation disrupts redox homeostasis by upregulating pro-oxidant enzymes, increasing reactive oxygen species (ROS) production, and reducing nitric oxide (NO) bioavailability, culminating in endothelial senescence and apoptosis. MR signalling also promotes pro-inflammatory and pro-fibrotic phenotypes in PAECs, further impairing vascular function. In pulmonary arterial smooth muscle cells (PASMCs), MR activation stimulates hyperproliferation, excessive extracellular matrix (ECM) accumulation, and perivascular inflammation. Together, these mechanisms drive pulmonary vascular remodelling and contribute to the progression of PAH [36].

### 5.1. Preclinical Studies

Finerenone has generated notable interest in this context. In a 2022 experimental study, finerenone partially reversed established pulmonary hypertension in rodent models [37]. The drug improved hemodynamics, reduced vascular remodelling, and attenuated inflammation even when initiated after disease onset, reinforcing the rationale for MR blockade as a therapeutic approach in PAH. The authors further demonstrated MR overexpression in human PAH lung tissue and showed that MR inhibition exerts direct antiproliferative effects in patient-derived pulmonary arterial smooth muscle cells, highlighting strong translational relevance.

However, the therapeutic effects observed were partial rather than fully restorative, and the reliance on monocrotaline and Sugen/hypoxia models—despite their utility—does not fully capture the complexity of human PAH. In addition, the absence of clinical data leaves important uncertainties regarding long-term safety, optimal dosing, and efficacy in patients. Even so, these findings position finerenone as a promising next-generation MRA that merits further evaluation, particularly as a potential add-on to existing PAH therapies.

Boehm et al. conducted an animal study using both monocrotaline and SU5416/hypoxia models of pulmonary arterial hypertension [38]. Their findings showed that eplerenone favourably influenced pulmonary vascular remodelling and reduced right ventricular systolic pressure. However, it did not produce significant improvements in right ventricular hypertrophy or fibrosis. These findings suggest that eplerenone may exert predominantly pulmonary vascular rather than myocardial effects. Complementary in vitro experiments further demonstrated direct anti-proliferative effects of MR blockade in human pulmonary artery smooth-muscle cells. More recent experimental evidence suggests that spironolactone may also exert direct effects on the pressure-overloaded right ventricle. In a PAH model, spironolactone improved right ventricular remodelling and was associated with suppression of myocardial steroidogenic acute regulatory protein expression, a regulator of local aldosterone synthesis [39]. These findings raise the possibility that mineralocorticoid receptor blockade may act not only on the pulmonary vasculature but also on maladaptive right ventricular remodelling. Nevertheless, the mechanistic findings remain experimental and require confirmation in human PAH.

Lastly, the preclinical work by Preston et al. is also noteworthy. Their study demonstrated that MR antagonism with spironolactone markedly attenuated experimental pulmonary hypertension in both hypoxia- and monocrotaline-induced models, leading to reductions in RV systolic pressure, pulmonary vascular resistance, and pulmonary vascular remodelling [40]. Notably, the therapeutic effect was most substantial when treatment was initiated early in the disease course and diminished once PAH was fully established, supporting the notion that MR blockade primarily influences vascular remodelling rather than providing late-stage RV rescue. The investigators further identified MR expression and activation in distal PASMCs and showed that spironolactone inhibited PASMC proliferation triggered by aldosterone, hypoxia, or PDGF. Taken together, these animal studies strongly support the aldosterone–MR axis as a compelling therapeutic target in group 1 PAH and provide a physiological rationale for clinical evaluation of MR antagonists in this patient population.

### 5.2. Clinical Data

Clinical evidence for MR antagonism in PAH remains limited and somewhat inconsistent. A retrospective analysis from the ARIES-1 and ARIES-2 trials showed a trend toward improved 6 min walk distance (6-MWD) and lower circulating B-type natriuretic peptide (BNP) levels in patients receiving spironolactone alongside ambrisentan (an endothelin-A receptor antagonist), compared with ambrisentan alone [41]. Notably, spironolactone use was associated with a greater reduction in circulating inflammatory markers than PAH-specific therapies. A separate retrospective study reported that MRA use correlated with greater disease severity among PAH patients, likely reflecting the tendency to prescribe MRAs selectively to those with more advanced symptoms [42].

However, the observational evidence is difficult to interpret because spironolactone is commonly prescribed to patients with more advanced right ventricular failure and fluid retention. In a pooled analysis of 1229 participants from pivotal PAH trials, patients receiving spironolactone had more severe disease at baseline and experienced higher rates of clinical worsening and mortality. These associations were attenuated after adjustment for disease severity, supporting substantial confounding by indication rather than a direct adverse effect of spironolactone [43]. Similar limitations apply to broader pulmonary hypertension cohorts, in which mineralocorticoid receptor antagonist use has been associated with worse outcomes largely among patients with advanced cardiopulmonary disease [42,44]. This issue is particularly relevant given the increasing prevalence of significant cardiopulmonary comorbidities among patients with PAH [45,46]. In one of these studies, the association between MRA therapy and increased mortality was attenuated after adjusting for disease severity, suggesting confounding by indication, as MRAs were preferentially prescribed to patients with more severe illness [44]. Prospective evidence has also been inconclusive. In a small randomized crossover study, spironolactone did not significantly alter circulating markers of collagen turnover or improve six-minute walk distance over 16 weeks, although it was generally well tolerated and was not associated with clinically significant hyperkalemia or liver-function abnormalities [47]. The limited sample size, short treatment duration, and relatively preserved baseline exercise capacity reduce the ability of this study to exclude a clinically meaningful effect in selected PAH phenotypes.

Taken together, these findings require cautious interpretation. In clinical practice, MRAs are frequently prescribed to patients with advanced right ventricular dysfunction and fluid retention, making it difficult to separate their therapeutic effects from the greater baseline risk of the patients who receive them. A randomized placebo-controlled phase 2 study was designed to evaluate spironolactone earlier in the course of PAH (NCT01712620), with change in six-minute walk distance as the primary outcome and additional assessment of right ventricular function, endothelial dysfunction, inflammatory markers, and safety [48]. However, no peer-reviewed efficacy results from this trial were identified at the time of the present review. Consequently, the available clinical evidence remains insufficient to determine whether MR blockade offers disease-modifying benefit beyond its established role in managing fluid retention.

In summary, elevated aldosterone levels in PAH have been associated with adverse haemodynamic and functional parameters in the absence of left-sided heart failure. MR activation in pulmonary vascular cells promotes oxidative stress, endothelial dysfunction, and vascular remodelling, all of which contribute to disease progression. These observations provide a strong mechanistic rationale for MR antagonism as a therapeutic strategy in PAH. However, robust clinical trials are still required to determine its true efficacy and safety in this population. A summary of the principal studies evaluating MRAs in pulmonary hypertension is presented in Table 2.

## 6. Discussion

Despite major advances in PAH-targeted therapies, PAH remains a progressive disease associated with substantial morbidity and mortality, particularly in patients with advanced right ventricular dysfunction [1]. Although current therapeutic strategies primarily focus on pulmonary vasodilation through nitric oxide, endothelin, and prostacyclin pathways, persistent vascular remodelling, endothelial dysfunction, inflammation, fibrosis, and metabolic dysregulation continue to contribute significantly to disease progression [17]. Consequently, adjunctive therapies targeting multiple pathophysiological pathways simultaneously remain an important unmet clinical need [4].

Importantly, right ventricular heart failure represents the final common pathway and the leading determinant of clinical deterioration and death in PAH [14,16]. Therefore, potential adjunctive therapies should not be evaluated solely according to their effects on pulmonary vascular resistance or pulmonary arterial pressure but also according to their impact on right ventricular adaptation, right ventricular–pulmonary arterial coupling, congestion, myocardial fibrosis, and energetic reserve [14]. In this context, SGLT2 inhibitors and MRAs are particularly relevant because their proposed mechanisms may extend beyond the pulmonary vasculature and involve direct or indirect effects on right ventricular remodelling, metabolism, inflammation, fibrosis, and volume homeostasis [19,36]. This is especially important in patients with established or evolving right ventricular dysfunction, in whom therapies that support right ventricular adaptation may provide clinically meaningful benefit even if their direct pulmonary vascular effects are modest.

In this context, SGLT2is have emerged as promising therapeutic agents due to their pleiotropic cardiovascular effects extending beyond glycemic control [49,50]. Experimental and early clinical evidence suggests that SGLT2i may improve pulmonary vascular remodelling, endothelial function, oxidative stress, mitochondrial dysfunction, and inflammatory signalling while additionally exerting beneficial effects on RV remodelling and myocardial energetics. These mechanisms are particularly relevant in PAH, where maladaptive RV remodelling and progressive pulmonary vascular disease are major determinants of prognosis. Furthermore, the growing recognition of cardiometabolic comorbidities in PAH populations strengthens the rationale for investigating SGLT2i in this setting [45].

Similarly, MRAs may provide additional therapeutic benefit through anti-inflammatory, antifibrotic, and antiproliferative mechanisms [51]. Aldosterone-mediated signalling has been implicated in pulmonary vascular remodelling, endothelial dysfunction, and RV fibrosis, suggesting that MRAs may exert beneficial pulmonary vascular and myocardial effects beyond their established diuretic properties [36]. Newer nonsteroidal MRAs, such as finerenone, may further expand these therapeutic possibilities due to their more selective receptor profile and potentially improved safety characteristics [51].

Although SGLT2 inhibitors and MRAs share several potentially relevant effects, their proposed roles in PAH are not identical. SGLT2 inhibitors may act predominantly through metabolic, mitochondrial, endothelial, anti-inflammatory, and haemodynamic pathways, with possible benefits extending to right ventricular energetics and cardiometabolic comorbidities. In contrast, MRAs more directly target aldosterone-mediated oxidative stress, fibrosis, vascular smooth-muscle-cell proliferation, and sodium and fluid retention. The evidence supporting both drug classes remains predominantly preclinical, although the experimental evidence for MR blockade is more directly linked to the aldosterone–mineralocorticoid receptor axis in PAH, whereas much of the human evidence for SGLT2 inhibition is extrapolated from diabetes and left-heart failure populations. These differences suggest that the two classes should not be regarded as interchangeable and may ultimately prove relevant to different PAH phenotypes or stages of disease.

The emergence of sotatercept and other highly effective PAH-specific therapies also changes the clinical question surrounding SGLT2 inhibitors and MRAs [8,9,10]. Rather than being viewed as competing alternatives to established PAH-targeted treatment, these agents may be better conceptualized as potential phenotype-directed adjunctive therapies. If sotatercept becomes widely incorporated into PAH treatment algorithms, the incremental role of SGLT2 inhibitors and MRAs will need to be tested against a higher background standard of care [1]. Their potential value may lie in complementary effects on right ventricular remodelling, metabolic dysfunction, renal protection, systemic inflammation, congestion, and coexisting cardiovascular disease, rather than in direct replacement of PAH-specific vasodilator or antiproliferative therapies [36].

Another unresolved question is whether combined SGLT2 inhibition and mineralocorticoid receptor blockade may offer additive benefit in PAH. In heart failure and chronic kidney disease, these two drug classes are frequently used together because they target partially distinct and potentially complementary pathways, including sodium handling, neurohormonal activation, inflammation, fibrosis, renal protection, and myocardial remodelling [18,19,36]. However, no dedicated clinical trial has evaluated combined SGLT2 inhibitor and MRA therapy in hemodynamically confirmed Group 1 PAH. At present, any discussion of this combination in PAH remains extrapolative. Future studies should assess whether combined therapy provides incremental benefit over either class alone, particularly in patients with right ventricular dysfunction, fluid retention, cardiometabolic comorbidities, or renal vulnerability. Safety evaluation will also be essential, given the potential for hypotension, excessive diuresis, renal dysfunction, electrolyte disturbances, and increased treatment burden in patients already receiving complex PAH-specific regimens.

## 7. Limitations and Future Perspectives

The current evidence supporting the use of SGLT2 inhibitors and MRAs in PAH remains limited by several important methodological and translational constraints. Most mechanistic and efficacy data derive from animal models that reproduce only selected features of human PAH and do not fully capture the heterogeneity, chronicity, comorbidity burden, or treatment background of contemporary patients. Even within preclinical studies, findings have not been uniform across different agents, doses, treatment timings, and experimental models, making it difficult to establish whether the observed effects represent true class effects. Human evidence is substantially weaker and consists largely of retrospective analyses, small prospective studies, or extrapolation from populations with diabetes, left-heart failure, or other forms of pulmonary hypertension. These data are therefore vulnerable to confounding, particularly confounding by indication in studies of MRAs, and cannot establish disease-modifying efficacy in hemodynamically confirmed Group 1 PAH. In addition, most available studies have focused on surrogate haemodynamic, biomarker, or imaging outcomes rather than clinical worsening, hospitalization, transplantation, or mortality.

Future trials should therefore adopt a more rigorous and phenotype-oriented approach. Randomized, adequately powered studies are required to evaluate SGLT2 inhibitors and MRAs as adjunctive therapies on top of contemporary PAH-specific treatment, with careful stratification according to PAH subtype, right ventricular function, renal function, volume status, metabolic profile, and background therapy. Potential responder phenotypes may include patients with cardiometabolic comorbidities, impaired right ventricular energetics, fluid retention, elevated aldosterone activity, or evidence of inflammatory and fibrotic activation. Trial endpoints should extend beyond six-minute walk distance and include invasive pulmonary hemodynamics, right ventricular–pulmonary arterial coupling, cardiac magnetic resonance indices, biomarkers, quality of life, time to clinical worsening, and safety outcomes. Particular attention should be paid to volume depletion, hypotension, renal dysfunction, electrolyte disturbances, genital infections, and potential interactions with diuretics and established PAH therapies. Mechanistic substudies incorporating circulating biomarkers, tissue-based analyses, and metabolic or imaging phenotyping may help determine whether these agents act primarily through pulmonary vascular, right ventricular, renal, or systemic pathways. Until such evidence becomes available, both drug classes should be considered investigational in PAH and should not be used with the expectation of disease modification outside clinical trials. Finally, as PAH-specific therapies improve survival, the treated PAH population is likely to become older and more comorbid, with increasing rates of systemic hypertension, diabetes mellitus, chronic kidney disease, obesity, atrial arrhythmias, and left-sided cardiac disease. Polypharmacy will therefore become an increasingly important clinical challenge, with implications for drug–drug interactions, tolerability, adherence, and treatment burden [45]. Fixed-dose combination strategies are already being developed for PAH-specific therapy, including endothelin receptor antagonist–phosphodiesterase type 5 inhibitor combinations [1,11], and similar approaches are being explored for non-steroidal MRAs and SGLT2 inhibitors in chronic kidney disease and heart failure [18,19,51]. Although such strategies have not yet been evaluated in PAH, they may become relevant if adjunctive cardiometabolic therapies are incorporated into the care of selected PAH phenotypes.

## 8. Conclusions

Pulmonary arterial hypertension remains a severe and progressive disease despite major advances in targeted therapies. Emerging evidence suggests that sodium–glucose cotransporter-2 inhibitors and mineralocorticoid receptor antagonists may exert beneficial pulmonary vascular and right ventricular effects through anti-inflammatory, antifibrotic, metabolic, endothelial, and volume-regulatory pathways. However, current evidence remains insufficient to support their routine use as PAH-directed therapies. In the contemporary era of increasingly effective PAH-specific treatments, including sotatercept, the most plausible role of SGLT2 inhibitors and MRAs may be as phenotype-guided adjunctive therapies, particularly in patients with right ventricular dysfunction, congestion, renal vulnerability, or cardiometabolic comorbidities. Combined SGLT2 inhibitor–MRA therapy is mechanistically attractive but remains untested in hemodynamically confirmed Group 1 PAH. Dedicated randomized clinical trials are required to clarify whether these agents provide incremental benefit on top of modern PAH-specific therapy.

## Figures and Tables

**Table 1:** Principal studies evaluating sodium–glucose cotransporter-2 inhibitors in pulmonary hypertension and related pulmonary haemodynamic settings.

Study	Study Design	Population/Model	Intervention	Main Findings	Relevance and Limitations
Kayano et al., 2020 [33]	Randomized controlled trial	Patients with type 2 diabetes and increased cardiovascular risk	Dapagliflozin	Reduced exercise-induced right ventricular (RV) systolic pressure and left ventricular filling pressure; no improvement in stroke volume index or cardiac index	Human haemodynamic evidence, but participants did not have confirmed pre-capillary PAH
Chowdhury et al., 2020 [30]	Experimental animal study	Monocrotaline-induced pulmonary hypertension	Empagliflozin	Reduced mortality, right ventricular systolic pressure, right ventricular hypertrophy and fibrosis, and pulmonary arterial muscularization	Supports pulmonary vascular and right ventricular effects, but findings are limited to an experimental model
Nassif et al., 2021, EMBRACE-HF [34]	Randomized placebo-controlled trial	Patients with heart failure monitored using implanted pulmonary artery pressure sensors	Empagliflozin	Reduced pulmonary artery pressures during follow-up	Indirect evidence from left-heart disease; changes may reflect reduced filling pressures or volume load rather than effects on pulmonary vascular remodelling
Li et al., 2021 [32]	Experimental animal study	Monocrotaline-induced PAH and pulmonary trunk-banding rat models	Dapagliflozin	No significant improvement in pulmonary hemodynamics, vascular remodelling, or right ventricular structure and function	Important negative study; suggests that beneficial effects may not represent a uniform class effect
Tang et al., 2022 [28]	Experimental animal study	Monocrotaline-induced PAH	Dapagliflozin, sildenafil, or their combination	Reduced right ventricular systolic pressure, pulmonary vascular remodelling, NLRP3 activation, and circulating interleukin (IL)-1β and interleukin-18; combination therapy was not superior to sildenafil alone	Supports anti-inflammatory and vascular effects but does not demonstrate additive benefit with established PAH therapy
Dabravolski et al., 2022 [29]	Mechanistic review	Cardiovascular cell and animal models	SGLT2 inhibitors	Described potential improvements in mitochondrial function, redox balance, and cellular survival	Mechanistically relevant, but evidence is not specific to human PAH
Youssef et al., 2023 [22]	Mechanistic review	Experimental cardiovascular and metabolic studies	SGLT2 inhibitors	Summarized anti-inflammatory, antioxidative, and anti-remodelling effects	Provides general mechanistic support rather than direct PAH evidence
Luo et al., 2024 [21]	Narrative review	Experimental pulmonary hypertension studies	SGLT2 inhibitors	Summarized potential improvements in pulmonary vasodilation, endothelial function, and vascular remodelling	Useful synthesis but not a primary experimental study
Zhang et al., 2025 [26]	Mechanistic review	Pulmonary vascular cell and experimental PH models	SGLT2 inhibitors	Suggested favourable effects on endothelial cells, pulmonary arterial smooth-muscle cells, inflammation, and vascular remodelling	Supports biological plausibility, but clinical evidence in Group 1 PAH remains limited
Lyu et al., 2026 [31]	Experimental animal and cellular study	Experimental pulmonary hypertension and pulmonary arterial smooth-muscle cells	Empagliflozin	Attenuated pulmonary vascular remodelling and smooth-muscle-cell proliferation through inhibition of PDGF receptor-β phosphorylation	Provides a specific antiproliferative mechanism, but human validation is lacking

HF, heart failure; IL, interleukin; NLRP3, NOD-, LRR- and pyrin domain-containing protein 3; PAH, pulmonary arterial hypertension; PDGF, platelet-derived growth factor; PH, pulmonary hypertension; RV, right ventricular; SGLT2, sodium–glucose cotransporter 2.

**Table 2:** Principal studies evaluating mineralocorticoid receptor antagonists in pulmonary hypertension.

Study	Study Design	Population/Model	Intervention	Main Findings	Relevance and Limitations
Preston et al., 2013 [40]	Experimental animal and cellular study	Hypoxia- and monocrotaline-induced pulmonary hypertension (PH) models and pulmonary arterial smooth-muscle cells	Spironolactone	Reduced right ventricular (RV) systolic pressure, pulmonary vascular resistance (PVR), and pulmonary vascular remodelling; inhibited pulmonary arterial smooth-muscle-cell proliferation induced by aldosterone, hypoxia, and platelet-derived growth factor (PDGF)	Supports a direct role of the aldosterone–mineralocorticoid receptor axis in pulmonary vascular remodelling; effects were greater when treatment was initiated early and remain unconfirmed in humans
Maron et al., 2013, ARIES-1/2 [41]	Retrospective analysis of clinical-trial data	Patients with PAH receiving ambrisentan	Spironolactone plus ambrisentan versus ambrisentan alone	Spironolactone use was associated with trends toward greater improvement in 6 min walk distance and reduction in B-type natriuretic peptide (BNP) and inflammatory markers	Provides hypothesis-generating clinical evidence, but the analysis was retrospective, underpowered, and susceptible to confounding
Elinoff et al. 2013 [48]	Randomized placebo-controlled phase 2 trial protocol	Patients with PAH	Spironolactone versus placebo	Designed to assess exercise capacity, right ventricular function, endothelial dysfunction, inflammatory biomarkers, and safety	Represents a dedicated prospective evaluation of spironolactone in PAH; peer-reviewed efficacy results were not identified at the time of this review
Boehm et al., 2018 [38]	Experimental animal and ex vivo study	Monocrotaline- and SU5416/hypoxia-induced PAH models and human pulmonary arterial smooth-muscle cells	Eplerenone	Attenuated pulmonary vascular remodelling and modestly reduced right ventricular systolic pressure; did not significantly improve right ventricular hypertrophy or fibrosis; inhibited smooth-muscle-cell proliferation	Suggests predominantly pulmonary vascular rather than direct right ventricular effects; evidence remains limited to experimental models
Andersson et al., 2019 [44]	Nationwide retrospective registry study	Patients with chronic obstructive pulmonary disease and right-sided heart failure	Mineralocorticoid receptor antagonists	MRA use was initially associated with increased mortality, but the association was attenuated after adjustment for disease severity	Findings are strongly affected by confounding by indication and were derived mainly from Group 3 pulmonary hypertension rather than Group 1 PAH
Safdar et al., 2020 [47]	Randomized crossover trial	Patients with PAH	Spironolactone	Spironolactone was generally well tolerated but did not significantly improve 6 min walk distance or circulating markers of collagen turnover over 16 weeks	Provides prospective human safety data, but the small sample size, short follow-up, and relatively preserved baseline exercise capacity limited the ability to detect clinical benefit
Lahm et al., 2021 [42]	Retrospective cohort study	Patients with pulmonary hypertension of mixed etiology, predominantly Group 3 disease	Renin–angiotensin–aldosterone system inhibitors, including MRAs	Use of renin–angiotensin–aldosterone system inhibitors was associated with clinical outcomes that varied according to pulmonary hypertension phenotype and baseline disease severity	Indirect evidence with limited applicability to Group 1 PAH; residual confounding and heterogeneous drug exposure prevent conclusions regarding an MRA-specific effect
Safdar and Cho, 2021 [43]	Pooled retrospective analysis of pivotal PAH trials	1229 patients with PAH	Spironolactone use versus no spironolactone	Patients receiving spironolactone had more severe disease and higher unadjusted rates of clinical worsening and mortality; associations were attenuated after adjustment for baseline severity	Illustrates substantial confounding by indication because spironolactone was preferentially prescribed to patients with advanced right ventricular failure and fluid retention
Tu et al., 2022 [37]	Experimental animal, human-tissue, and in vitro study	Monocrotaline- and SU5416/hypoxia-induced pulmonary hypertension models, human PAH lung tissue, and patient-derived pulmonary arterial smooth-muscle cells	Finerenone	Partially reversed established pulmonary hypertension, improved hemodynamics, reduced vascular remodelling and inflammation, and exerted direct antiproliferative effects	Provides strong translational support for selective MR blockade, but efficacy was partial and clinical safety, dosing, and effectiveness remain unknown
Mamazhakypov and Lother, 2023 [36]	Mechanistic review	Pulmonary vascular and cardiac cellular and experimental models	Mineralocorticoid receptor antagonists	Summarized evidence that MR activation promotes oxidative stress, endothelial dysfunction, inflammation, fibrosis, and pulmonary vascular remodelling	Provides a comprehensive mechanistic framework but does not constitute primary experimental or clinical evidence
Imano et al., 2024 [39]	Experimental animal study	Experimental PAH with pressure-overloaded right ventricular remodelling	Spironolactone	Improved right ventricular remodelling and suppressed myocardial steroidogenic acute regulatory protein expression, a regulator of local aldosterone synthesis	Suggests a possible direct myocardial effect of MR blockade, but the findings remain preclinical and require validation in human PAH

BNP, B-type natriuretic peptide; MRA, mineralocorticoid receptor antagonist; MR, mineralocorticoid receptor; PAH, pulmonary arterial hypertension; PDGF, platelet-derived growth factor; PH, pulmonary hypertension; PVR, pulmonary vascular resistance; RV, right ventricular; SU5416, vascular endothelial growth factor receptor 2 inhibitor.

## Data Availability

No new data were created or analyzed in this study. Data sharing is not applicable to this article.

## Abbreviations

BNP	B-type natriuretic peptide
CCB	Calcium channel blocker
CKD	Chronic kidney disease
ECM	Extracellular matrix
ERA	Endothelin receptor antagonist
ET-1	Endothelin-1
HF	Heart failure
HIV	Human immunodeficiency virus
ICAM-1	Intercellular adhesion molecule 1
IL	Interleukin
MMP-9	Matrix metalloproteinase 9
MR	Mineralocorticoid receptor
MRA	Mineralocorticoid receptor antagonist
NLRP3	NOD-, LRR- and pyrin domain-containing protein 3
NO	Nitric oxide
PAEC	Pulmonary arterial endothelial cell
PAH	Pulmonary arterial hypertension
PASMC	Pulmonary arterial smooth-muscle cell
PDE5i	Phosphodiesterase type 5 inhibitor
PDGF	Platelet-derived growth factor
PH	Pulmonary hypertension
PVR	Pulmonary vascular resistance
RAAS	Renin–angiotensin–aldosterone system
ROS	Reactive oxygen species
RV	Right ventricle/right ventricular
SGLT2	Sodium–glucose cotransporter 2
SGLT2i	Sodium–glucose cotransporter 2 inhibitor
SU5416	Vascular endothelial growth factor receptor 2 inhibitor
T2DM	Type 2 diabetes mellitus
TNF-α	Tumour necrosis factor alpha
VCAM-1	Vascular cell adhesion molecule 1
VEGF	Vascular endothelial growth factor
